# The neural impacts of gun violence exposure on adult attentional mechanisms: an EEG pilot study

**DOI:** 10.1080/28324765.2026.2643551

**Published:** 2026-03-15

**Authors:** Emma F. Ehrenzeller, Paul J. Smith, Timothy O. Mousseau, Girija Chatufale, Sarah A. Bennett, Michela N. Thomsen, Kay James

**Affiliations:** a Department of Biobehavioral Sciences, Teachers College, Columbia University, New York, NY, USA; b Students’ Educational and Cultural Movement of Ladakh [SECMOL] School, Leh, UT Ladakh, India; c Department of Child & Adolescent Psychiatry, NYU Grossman School of Medicine, New York, NY, USA; d Department of Clinical Psychology, William James College, Boston, Massachusetts, USA

**Keywords:** Gun violence, firearm-related harms, event related potentials, neural impacts of gun violence, attentional allocation

## Abstract

Adolescents and young adults experience a disproportionately high risk of gun violence victimization while also undergoing a sensitive neurodevelopmental period for cognitive processes like attention. Consequently, in this exploratory study, we evaluated the impact of gun violence exposure (GVE) on neural indices of attentional systems in late adolescents and young adults. We hypothesized that GVE decreases efficiency of the primary attentional mechanisms in the brain: alerting, orienting, and executive control. We recorded electroencephalography while participants completed the Attention Network Test (ANT) to elicit N1, N2, and P3 event-related potentials (ERPs), indices of alerting, orienting, and executive control. Participants provided self-reports of mental health and violence exposure. Behavioral and ERP differences between participants with and without GVE were evaluated. Results from 13 participants (ages 18–35; *M* = 25.54; *SD* = 4.16) demonstrated that participants with GVE experienced less efficiency in the attentional alerting mechanism compared to participants with no GVE. These findings may relate to hypervigilance associated with anxiety disorders that can arise following traumatic firearm exposure. Preliminary evidence for the disruptive effects of GVE on the neural correlates of attention indicates a need for more robust research to understand the effects of firearm-related harms on neural and cognitive functions.

## Introduction

Gun violence victimization, broadly defined as the experience of being threatened or directly harmed with a firearm, most severely affects teenagers and young adults from 15−34 years old (Centers for Disease Control and Prevention, 2022). Developmentally, this age range overlaps with late adolescence, which spans from 18−21 years (American Psychological Association, n.d.), with some evidence indicating that this stage of neurobiological development continues until age 24 (Sawyer et al., 2018). During this period, neuroplastic reorganization takes place to refine neural connections related to higher order functions, such as emotional regulation, inhibitory control, and attentional allocation (e.g. Giedd, 2004, 2008). Though highly plastic and resilient, the brain is also vulnerable during such periods of reorganization: exposure to various forms of trauma, such as gun violence, can alter neurodevelopmental trajectories and impact emergent cognitive functions. These trajectories may include the development of neural regions associated with emotion regulation (see, e.g. Gard et al., 2022) as well as the additional functions supported by those regions, such as vigilance and attention (e.g. McCoy et al., 2015).

Presently, there is a dearth of literature probing specific neural and cognitive effects of GVE. Neuroscience provides methods by which we can interrogate the physiological changes that may underpin behavior or precede behavioral changes. For example, neuroimaging approaches could be applied to investigate the neural changes that occur prior to or concurrently with the manifestation of psychiatric symptoms or altered cognitive function following GVE. Tools like functional magnetic resonance imaging (fMRI) can allow researchers to better understand how regions of the brain, or their connections, may differentially develop due to this exposure. Other methods, such as electroencephalography (EEG), can track how the brain’s immediate processing of various stimuli may shift with increased GVE. EEG and fMRI represent only two of many potential avenues to apply a neuroimaging approach to studying the impacts of gun violence. More broadly, pairing neuroscientific research with traditional psychological and psychosocial measures could develop a multi-dimensional picture of the effects of GVE, beyond behavior change.

Neuroscience research has only recently been applied to explore how exposure to gun violence may affect neural development and function in adolescents and young adults. For instance, Gard et al. (2022) provided evidence that living in proximity to fatal homicides may weaken connectivity between medial prefrontal cortex and amygdala in adolescents (*n* = 237; ages 15−17 years). Decreased connectivity between these regions can negatively impact emotional regulation, management of aggressive behavior, and allocation of appropriate attentional responses to potential threats (Park et al., 2018; Tottenham & Galvan, 2016). Parra et al. (2022) evaluated adrenocortical responses (indices of stress reactivity that can be measured via salivary samples) of California-based young adults (*n* = 202; ages 18−29) in the weeks following the Pulse Nightclub Massacre in Orlando, Florida. They concluded that even vicarious exposure to firearm-related tragedies may increase adrenocortical responsivity, particularly in individuals who do not typically experience high levels of stress in their daily lives. Neuropsychiatric evidence has also indicated strong connections between various mental health disorders and GVE for adolescents and adults: increased GVE has been associated with increased scores on measures assessing post-traumatic stress disorder (PTSD; Allwood et al., 2023; Turner et al., 2019; Vella et al., 2020), depression (Leibbrand et al., 2021; Magee et al., 2022), and anxiety (Leibbrand et al., 2020; Magee et al., 2022; Shulman et al., 2021). These neuropsychiatric differences may underlie negative effects on academic engagement and achievement, which have been evidenced in relation to both community violence exposure (Borofsky et al., 2013) and direct in-school gun violence experiences (Strøm et al., 2016).

One primary cognitive function, attention, plays a dominant role in the functionality of anxiety disorders highly associated with GVE, particularly the hypervigilance associated with PTSD (American Psychiatric Association, 2013). Individuals with trauma exposure and PTSD show disruptions to attentional capacities, especially heightened physiological arousal (Naegeli et al., 2018) alongside impaired processing of attentional cues that incorporate spatial information (Russman Block et al., 2020). Attentional processing is a key cognitive function primarily supported by the prefrontal cortex, which does not reach maturation until late adolescence (Lenroot & Giedd, 2006; Sowell et al., 2003). Against this background, attentional processing becomes an apt domain for the exploration of neural impacts associated with varying levels of gun violence exposure.

### Attention in the brain

At the neural level, attention is conceptualized through a framework proposed by Posner and Petersen (1990), which delineates three systems of attentional processing: alerting, orienting, and executive control. Alerting is the ability to initiate a state of arousal in response to a stimulus (Fan et al., 2002); as a practical example, alerting occurs when sirens blare nearby and the nervous system alters its state of arousal accordingly. Alerting is supported by frontal and parietal regions generally lateralized to the right hemisphere (Petersen & Posner, 2012; Posner & Petersen 1990). While alerting is about being prepared and vigilant, orienting directs attentional resources to specific targets or locations. Continuing the example, orienting occurs when you turn your head in the direction of the sirens. Sensory networks are crucially involved in orienting responses; for example, visual areas like the primary visual cortex and extrastriate regions support orienting to visual stimuli (Petersen & Posner, 2012). By contrast, executive control of attention encompasses multiple cognitive functions, including response inhibition (the capacity to withhold a reaction; e.g. Mostofsky & Simmonds, 2008) and conflict resolution (the ability to detect and respond to incongruence between stimuli; e.g. Fan et al., 2002). One application of executive control to the sirens example could be if sirens were identified as coming from opposite directions, resulting in the immediate need to determine a plan of action incorporating opposing environmental cues. Multiple brain regions are engaged in executive attentional control, including the medial prefrontal cortex, anterior cingulate cortex, and insula, all of which have been associated with the ability to identify and respond to conflicting stimuli (e.g. Dosenbach et al., 2006; Petersen & Posner, 2012).

#### Measuring attention

The primary behavioral paradigm to assess the function of these three systems is the Attention Network Test (ANT; Fan et al., 2002). We describe this paradigm in more detail below; to summarize, participants are presented with a cue (asterisk) that indicates the presence of an upcoming target (series of five arrows arranged horizontally). Depending on the condition, the center arrow may point in the same or opposite direction as the other arrows. Additionally, the cue may not appear for a given trial, or may appear with some spatial information on where the arrows will appear. These combinations of different conditions allow for independent determination of the responsivity of these three primary attentional systems.

The ANT can be paired with concurrent electroencephalography (EEG), a neuroimaging technique that measures electrical signals generated by the outer layers (cortex) of the brain (e.g. Woodman, 2010). To determine what specific activations are associated with the experimental manipulations, continuous EEG recordings are segmented to permit identification of signals that are time-locked to the onset of specific stimuli (such as the cue or targets of the ANT). These stimulus-locked fluctuations are averaged across similar conditions to elicit characteristic responses known as event-related potentials (ERPs; Luck, 2005). Specific ERP segments that have a similar polarity, scalp localization, amplitude, and timing are called components (Woodman, 2010); ERP components arise in response to stimuli and are understood as indices of specific neural functions. In this way, ERP data provides a method of assessing unique perceptive or attentional responses to different types of inputs and between various groups. The ERP technique has been paired with the ANT in a number of studies (see, e.g. Galvao-Carmona et al., 2014; Neuhaus et al., 2010, among others). Within these studies, three specific ERP components have been identified as particularly responsive to different conditions within the ANT: the N1, N2, and P3.

**N1.** The N1, also known as the N100, is the first negative deflection seen in an ERP waveform, typically arising between 80 and 200 ms post-stimulus. The N1 has been associated with various abilities related to attention, namely detecting visual stimuli and responding to their location in space (Correa et al., 2006; Griffin et al., 2002). Consequently, the N1 is associated with the orienting and alerting responses (Neuhaus et al., 2010), and it is typically observed over occipital and parietal regions in response to visual stimuli (Vogel & Luck, 2000). In the ANT paradigm, the N1 indexes an individual’s ability to initiate and direct their attention to cue (the asterisk) and target stimuli (the five arrows; Neuhaus et al., 2010).

**N2.** The N2 is a family of components with negative deflection generally manifesting between 200 and 350 ms post-stimulus. N2 signatures arising from frontal brain regions have been shown to correspond with the neural assessment of cognitive control and novelty (Folstein & Van Petten, 2008). More specifically, anterior N2 amplitudes increase in response to conflicting or incongruent stimuli, which is relevant to the executive control facet of attention (Folstein & Van Petten, 2008; Williams et al., 2016). The anterior cingulate cortex (ACC) is thought to be the origin of the frontal N2 component (Nieuwenhuis et al., 2003). As the ACC has been associated with error detection (for more, see, e.g. Botvinick et al., 2004), the N2 may reflect an individual’s detection of conflicting stimuli, such as the central arrow pointing in the opposite direction of the flanking arrows (Williams et al., 2016).

**P3**. The P3, also known as the P300, is a positive ERP component typically arising between 250 and 400 ms after the presentation of a stimulus. It frequently correlates with response inhibition and the management of conflicting stimuli required for executive control (Luck, 2005; Woodman, 2010), and enhanced difficulty of the attentional task has been shown to increase P3 amplitudes (Isreal et al., 1980). The P3 characteristically arises from frontal and parietal regions of the brain across a variety of paradigms (Luck, 2005; Neuhaus et al., 2010). During the ANT, the P3 is believed to represent the processing efficiency of an individual’s response to incongruent stimuli, such as the opposing central arrow (Neuhaus et al., 2010).

### Current study

As the current literature lacks investigations of the neural impacts of firearm-related harms, we set out to conduct an exploratory pilot study with two objectives: (1) to evaluate the feasibility of ERPs and the Attention Network Test (ANT; Fan et al., 2002) for multi-modality investigations of the attentional impacts of gun violence exposure; and (2) to identify patterns of difference in behavioral and neural indices of attentional processing in young adults affected by gun violence exposure. We hypothesized that GVE functions as a form of trauma and elicits a persistent state of hypervigilance, enhancing attentional alertness but ultimately reducing the efficacy of attentional processing by overtaxing relevant neural and cognitive resources. Based on this hypothesis, we predicted that participants with gun violence exposure would show impaired behavioral performance (reaction time and accuracy) and diminished peak ERP amplitudes in executive control and alerting conditions of the ANT compared to control participants.

## Methods

We recruited sixteen adult participants ages 18–35 (*M* = 25.44 years; *SD* = 4.94; 11 female-identifying, 5 male-identifying) into the study by means of sharing flyers on Teachers College’s online bulletin boards. Recruitment outreach via newsletters and other virtual communications was also conducted with gun violence advocacy groups such as Everytown for Gun Safety. Participants were screened for normal or corrected-to-normal vision; English proficiency; no diagnosis of ASD or ADHD; and no history of neurological trauma. All participants provided written, informed consent before initiation of research activities, and were compensated $80 for their contributions to the research. All study procedures were approved and conducted under the oversight of the Institutional Review Board at Teachers College, Columbia University (Protocol #23-128).

The final sample included 13 participants (*M* = 25.54 years; *SD* = 4.16 years; 9 female-identifying, 4 male-identifying) across the NGVE (*n* = 7) and GVE (*n* = 6) groups. Six participants indicated White and six indicated Asian as their primary race, and one participant indicated their ethnicity as Hispanic or Latino. Nine participants were undergraduate or graduate students, with three reporting full- or part-time employment and one reporting being unemployed. The highest level of education for participants’ caregivers was a master’s degree (*n* = 6). Annual income was reported as ʻUnder $15,000’ for the majority (*n* = 7) of participants. Participant demographics are shown in Table 1.

**Table 1.** Participant demographics.

Characteristic	All		NGVE		GVE	
	*n*	%	*n*	%	*n*	%
Gender						
Female	9	69	5	71	4	67
Male	4	31	2	29	2	33
Race						
White	6	46	2	29	4	67
Asian	6	46	4	57	2	33
Other (Unspecified)	1	8	1	14	0	0
Ethnicity						
Hispanic or Latino	1	7	1	14	0	0
Other (Unspecified)	1	7	1	14	0	0
Highest Education						
Bachelor’s	9	69	4	57	5	83
Master’s	3	23	3	43	0	0
Professional	1	7	0	0	1	17
Employment						
Student	9	69	5	71	4	67
Part-Time	1	8	1	14	0	0
Full-Time	2	15	0	0	2	33
Unemployed	1	8	1	14	0	0
Highest Parental Education						
Some High School	1	8	0	0	1	17
High School Diploma	1	8	1	14	0	0
Some College	0	0	0	0	0	0
Bachelor’s Degree	3	23	3	43	0	0
Master’s Degree	6	46	3	43	3	50
Professional Degree	2	15	0	0	2	33
Annual Income						
Under $15,000	7	54	4	57	3	33
$15,000−$24,999	1	8	0	0	1	17
$25,000−$34,000	2	15	2	29	0	0
$35,000−$49,000	0	0	0	0	0	0
$50,000−$74,999	1	8	0	0	1	17
$75,000−$99,999	0	0	0	0	0	0
$100,000−$149,000	1	8	0	0	1	17
$150,000+	1	8	1	14	0	0

Note: Participant demographics, including gender, race, ethnicity, highest education level, employment status, highest parental education status, and annual income, are shown for all participants, the no gun violence exposure (NGVE) group, and gun violence exposure (GVE) group.

All participants in the GVE group reported hearing gunfire in their neighborhoods, and four of the six described multiple instances of violent firearm-related experiences. Three participants recounted participating in lockdown drills prompted by actual school shootings or threats of school violence. Other participants described experiences such as witnessing a civilian shoot multiple police officers and attending to patients with severe gunshot wounds in a trauma center. None of the participants in either group indicated firearm ownership. Participant community experiences are summarized in Appendix 1.

### Mental health & violence exposure measures

Study participants completed self-report questionnaires concerning their demographics, violence exposure, and mental health via Qualtrics. The Beck Depression Inventory-I (BDI-I; Beck et al., 1961) was completed to assess the presence and severity of depressive symptoms, and the Emotional Regulation Questionnaire (ERQ; Gross & John, 2003) was used to assess cognitive reappraisal and emotional suppression abilities. The reliability and validity of the ERQ has been demonstrated in adolescent and young adult samples (e.g. Gross & John, 2003; Wang et al., 2022) and broader samples of U.S. adults (e.g. Preece et al., 2021).

Participants also completed an adapted version of the Survey of Exposure to Community Violence (SECV; Richters & Saltzman, 1990), which is a 54-item self-report measure of an individual's victimization and witnessing of various forms of violence. Both the full and abridged versions of SECV have demonstrated acceptable to excellent internal consistency (Rosenthal & Wilson, 2003, 2006; Rosenthal, 2000) and concurrent validity with cumulative trauma experiences (Scarpa et al., 2002) for college-aged young adults. Acceptable internal consistency (Mitchell et al., 2009) and reliability (Putman et al., 2009) have been evidenced in diverse adult populations as well (for more, see DeCou & Lynch, 2017). For this study, the SECV was adapted to include fourteen questions regarding exposure to gun violence, illicit drugs, and assaults. Due to our unique focus on gun violence exposure, our questions do not correspond with a previously examined adaptation of the SECV. In the analysis, the five firearm-related questions from the SECV were isolated to create a firearm exposure subscale, and an optional free response question allowed participants to offer additional detail about any gun violence experiences.

The firearm-related subscale of the SECV was used to categorize participants into two groups. Participants who did not indicate any gun violence exposure on the SECV were categorized into a no gun violence exposure (NGVE) group. Participants who indicated a specific exposure to gun violence (e.g. ‘I have heard gunfire in my neighborhood’; ‘I have witnessed someone else being shot with a firearm’), or who detailed other exposure using the free response option, were added to the gun violence exposure (GVE) group. Reporting non-violent or vicarious exposure to firearms, including reading about gun violence in the media or owning guns, or exposure to non-firearm related violence, did not result in categorization to the GVE group. Nine participants were included in the NGVE group, and seven participants in the GVE group.

### Attention network test (ANT)

**Figure 1.**
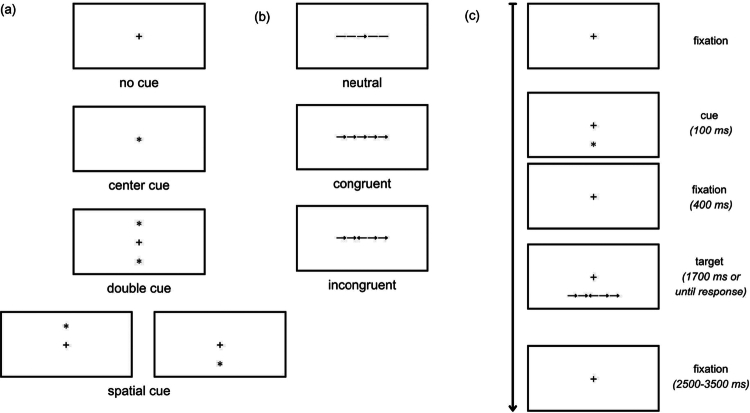
Experimental Procedure of the Attention Network Test (ANT). (a**).** Representation of cue trials. (b). Representation of target trials. (c). Portion of experimental timeline. Cues, targets, and paradigm adapted from Fan et al. (2002).

The ANT was presented to participants via E-Prime 3.0 (Psychology Software Tools, Inc.) following the design of Fan et al. (2002). Throughout the ANT, a fixation cross remained in the center of the screen unless a cue or target replaced it. The cue stimulus (an asterisk) could appear above or below the central fixation cross (Spatial Cue); simultaneously above and below the fixation cross (Double Cue); or by replacing the fixation cross in the middle of the screen (Center Cue). Cues appeared 500 ms before the target stimulus and remained on the screen for a duration of 100 ms. In some trials, no cues appeared (No Cue). Five horizontal arrows or lines comprised the target stimuli, which appeared above or below the fixation cross for 1700 ms or until the participant responded. Targets appeared in three configurations: all arrows pointing the same direction (congruent); the central arrow pointing in the opposite direction from the other arrows (incongruent); and center arrow flanked by lines rather than arrows (neutral). Participants were instructed to press the left- or right-most button on a response device to indicate the direction of the central arrow as quickly and accurately as possible. The intertrial interval ranged between 2500 and 3500 ms. Visual representations of the cues, targets, and experimental timeline are shown in Figure 1.

Following the design of the ANT by Fan et al. (2002), the entire experiment consisted of 288 trials split into three blocks of 96 trials. In each block, there were equal numbers of cue and target types, presented in a random order. All stimuli were presented to participants sitting 65 cm away from the computer monitor so that stimuli subtended 1.6–3.6 degrees of visual angle. The monitor was set at a brightness of 75 cd/m^2^.

The ANT permits assessment of individual attentional mechanisms (alerting, orienting, executive) by comparing the behavioral (reaction time, accuracy) and neural (EEG) response differences between specific cues and targets. Alerting is evaluated by the contrast between responses to Double Cue and No Cue conditions; orienting by contrasting spatial and central cue conditions; and executive control by contrasting congruent and incongruent target responses.

### EEG recording

For high-density EEG recording, participants were fitted with a 128-channel HydroCel Geodesic Sensor Net (Magstim Electrical Geodesics, Inc.). Data were collected via Net Station 4.3.1 using a Net Amps 200 series amplifier at a sampling rate of 500 Hz with an online vertex reference (Cz). During recording, a low-pass filter of 200 Hz and high-pass filter of .1 Hz were applied. Electrode impedances were measured after net placement and between trial blocks to ensure all impedances were maintained below 40 kΩ. All sessions were conducted in an electrically shielded and sound attenuated room.

After the electrode net was fitted, participants completed one practice round of the ANT in which feedback was given on the accuracy and speed of their responses. Participants then completed three trial blocks of the ANT while EEG data were recorded. The order of the Qualtrics questionnaire session and EEG data collection was counterbalanced between participants.

### Data pre-processing

EEG data analysis was performed with the Harvard Automated Pre-Processing Pipeline for Electroencephalography (HAPPE) 4.1 (Gabard-Durnam et al., 2018) in MATLAB. In HAPPE, a bandpass filter of 0.3−30 Hz was applied, and globally bad channels were automatically detected and removed. An average of 91.6% (SD: 3.7%, range: 86.0%−97.7%) of channels were retained across all participants. Artifact detection was completed using a hard wavelet threshold, which allows for the removal of artifacts from continuous EEG data while maintaining raw data. For the target-locked analysis, the raw data were segmented from the onset of the target stimuli. For all three conditions, a 700 ms time window was used, initiating 100 ms before the target appearance and ending 600 ms afterwards. For the cue-locked analysis, the onset of the target stimuli was still used as the zero point of the segment; however, the window was expanded to 600 ms before the target appearing to capture the onset of the cue 500 ms before the target.

Spherical spline interpolation of data from surrounding electrodes was used to replace bad channels. Data were re-referenced to the average response of all scalp electrodes. A baseline segment of 100 ms of prestimulus time was subtracted from the data; for the cue-locked analysis, this included the 100 ms prior to the cue. Segments were rejected if they fell outside of amplitude boundaries of -150 and 150 μV. Following data preprocessing in HAPPE, any participants with less than 40% usable trials were removed from the analysis. Three participants were removed based on this threshold (two NGVE, one GVE), leaving 13 participants (seven NGVE, six GVE) for subsequent analyses. On average, 63.80% (SD: 5.44%) of all trials were usable across these 13 participants.

### Statistical analysis

**Figure 2.**
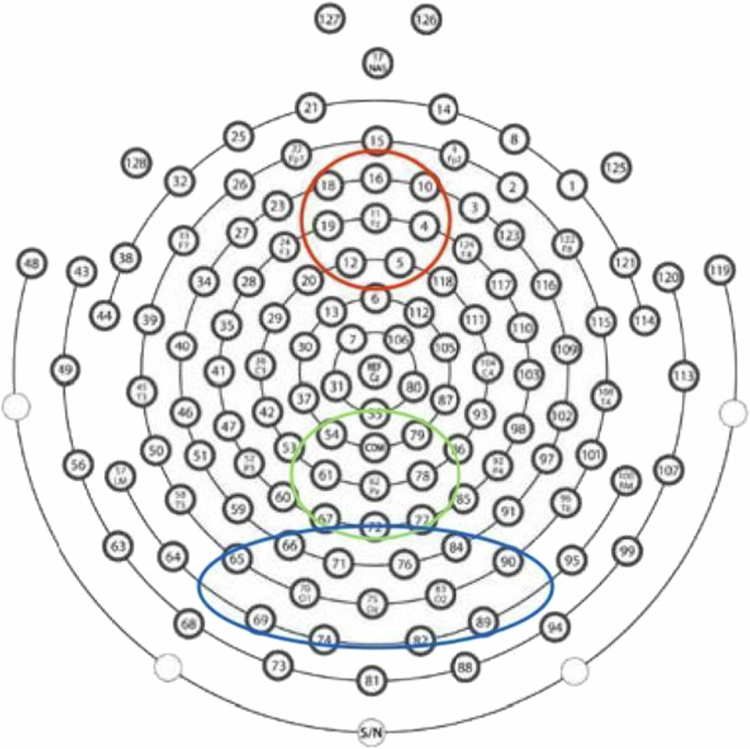
Montages of interest for our analysis. Frontal montage (red): electrodes 4, 5, 10, 11, 12, 16, 18, and 19. Parietal montage (green): electrodes 54, 61, 62, 67, 72, 77, 78, and 79. Occipital montage (blue): electrodes 65, 66, 69, 70, 71, 74, 75, 76, 82, 83, 84, 89, and 90.

To isolate and extract ERP components, montages (collections of electrodes) from across the scalp were selected based on their proximity to brain regions demonstrated to generate N1, N2, and P3 ERPs during the ANT procedure, per Galvao-Carmona et al. (2014) and Neuhaus et al. (2010). We included a frontal montage centered around Fz, a parietal montage centered around Pz, and a bilateral occipital montage centered around Oz. Specific electrodes included in each montage are indicated in Figure 2. For both cue-locked and target-locked analyses, the alerting N1 was computed within the parietal montage, the orienting N1 in the occipital montage, and the executive N2 and P3 in the frontal and parietal montages, respectively.

R Software (Version 4.4.2) was used to calculate adaptive means for each component in each participant, by extracting the largest value within the component-specific time window and averaging together amplitudes within 10 ms on either side. The time windows used to identify the adaptive means for target-locked N1, N2, and P3 components were 50−200 ms, 200−400 ms, and 250−450 ms post-target, respectively. For the cue-locked components (N1 and P3), similar time windows were used following the presentation of the cue. Grand averaged waveforms were generated for each group, over each montage, also in R.

Statistical analyses for behavioral data were conducted with IBM SPSS for Macintosh (Version 28.0.0.0). Descriptive statistics (mean, standard deviation) were calculated for RT and adaptive mean peak amplitudes for each condition, each group, and all participants. Independent samples one-tailed *t*-tests were used to assess differences on the BDI-I, ERQ-R/S, and SECV between the NGVE and GVE groups. Because the study had a small sample with repeated measures, a linear mixed model (LMM) was used instead of ANOVA since LMMs handle within-subject variability and missing data more flexibly while reducing the risk of biased results. Trial-level amplitudes were computed for each participant, and an LMM was constructed for each component, with Group, Condition and their interaction serving as fixed effects; a random intercept was allowed for each participant. All analyses were completed in R; models themselves were computed via restricted maximum likelihood estimation using the nlme package, and *p*-values were found using the lmerTest package and the Satterthwaite degrees of freedom correction. Significant main effects or interactions were followed up with pairwise comparisons applying a Bonferroni correction.

## Results

### Assessment scores

No significant differences were found between the GVE and NGVE groups on scores for the BDI-I, ERQ-R, or SECV. The GVE group scored significantly lower than the NGVE group on the ERQ-S (*p* = .05), and significantly higher on the firearm subscale of the adapted SECV (*p <* .001). Means and standard deviations of the group scores on these measures are shown in Table 2.

**Table 2.** Mental health questionnaire responses.

	All		NGVE		GVE	
Inventory	*M*	SD	M	SD	M	SD
BDI-I	8.8	6.6	7.0	4.4	11.0	8.7
ERQ-R	27.9	5.5	28.9	4.5	26.7	6.8
ERQ-S*	16.3	5.1	18.4	3.3	13.8	6.0
SECV	6.8	3.5	5.6	3.4	8.2	3.5
Firearms Subscale**	2.0	1.9	0.6	0.8	3.4	1.4

Note: The mean and standard deviation scores for each group and all participants are shown for the mental health and violence exposure measures. BDI: Beck Depression Inventory; ERQ-R: Emotional Response Questionnaire - Reappraisal; ERQ-S: Emotional Response Questionnaire - Suppression; SECV: Survey of Exposure to Community Violence [Adapted].

^*^ Denotes significantly different scores between GVE and NGVE groups at *p* < .05;

^**^ at *p* < .01.

### Behavioral data

Average accuracy values and reaction times for each group and condition are reported separately for each attentional network (alerting, orienting, executive) in Tables 3 and 4, respectively. Two-way mixed model ANOVA (Group x Condition) was evaluated for accuracy within each attentional network. No significant interaction effects or main effects were detected for accuracy in any of the attentional systems. Overall, accuracy was high for all conditions and across both groups.

**Table 3.** Accuracy for cue conditions and target types.

		NGVE	GVE
Condition	Stimuli	*M (SD)*	*M (SD)*
Alerting	No cue	99.40 (1.09)	99.77 (0.57)
	Double cue	99.01 (1.32)	99.77 (0.57)
Orienting	Center cue	98.61 (2.54)	98.84 (1.05)
	Spatial cue	99.21 (1.57)	99.77 (0.57)
Executive control	Congruent target	100 (0)	99.83 (0.43)
	Incongruent target	97.61 (2.06)	98.96 (1.14)

Note: Mean and standard deviation of ANT accuracy are shown for the alerting, orienting, and executive control conditions for the NGVE and GVE groups. Accuracy is shown as a percentage.

**Table 4.** Reaction time for cue conditions and target types.

		NGVE	GVE
Condition	Stimuli	*M (SD)*	*M (SD)*
Alerting**	No cue	532.65 (86.53)	540.75 (49.44)
	Double cue	487.81 (75.63)	504.1 (54.31)
Orienting**	Center cue	490.13 (80.89)	513.13 (49.91)
	Spatial cue	459.45 (56.74)	470.73 (52.69)
Executive Control**	Congruent target	477.16 (71.41)	482.38 (44.14)
	Incongruent target	538.75 (84.88)	565.02 (68.74)

Note: Mean and standard deviation reaction times are shown for the alerting, orienting, and executive control conditions for the NGVE and GVE groups. Times are shown in milliseconds.

^**^ Denotes significant main effect of Condition at *p* < .001.

Similarly for reaction time, two-way mixed model ANOVA (Group x Condition) was conducted within each attentional network. For the alerting conditions (No Cue vs. Double Cue), no significant Group x Condition interaction was detected (*F* (1, 11) = .471, *p* = .507, partial η^2^ = .041) and a main effect of Group was not found (*F* (1, 11) = .102, *p* = .756, partial η^2^ = .009); however, a main effect of Condition was (*F* (1, 11) = 46.735, *p* < .001, partial η^2^ = .809). Planned comparisons confirmed that reaction times to the No Cue trials were significantly longer than for the Double Cue trials (*M* = 40.745, *p* < .001).

For orienting trials (Center Cue vs. Spatial Cues), we found similar results: there were no significant Group x Condition interaction (*F* (1, 11) = 5.62, *p* = .469, partial η^2^ = .049) or main effect of Group (*F* (1, 11) = .259, *p* = .621, partial η^2^ = .023), though there was a significant main effect of Condition (*F* (1, 11) = 21.853, *p* < .001, partial η^2^ = .665). Participants showed significantly longer reaction times for trials with a central cue as compared to a Spatial Cue (*M* = 36.539, *p* < .001).

Lastly, results for the executive attention trials followed the same pattern, with a significant main effect of Condition (*F* (1, 11) = 81.020, *p* < .001, partial η^2^ = .880) but no significant Group x Condition interaction (*F* (1, 11) = 1.725, *p* = .216, partial η^2^ = .136) or main effect of Group (*F* (1, 11) = .172, *p* = .687, partial η^2^ = .015). Congruent trials had significantly faster reaction times (*M* = −72.114, *p* < .001). These results generally align with traditional reaction time comparisons for the Attention Network Task (e.g. Fan et al., 2002; Neuhaus et al., 2010).

### Target−locked analyses

**Figure 3.**
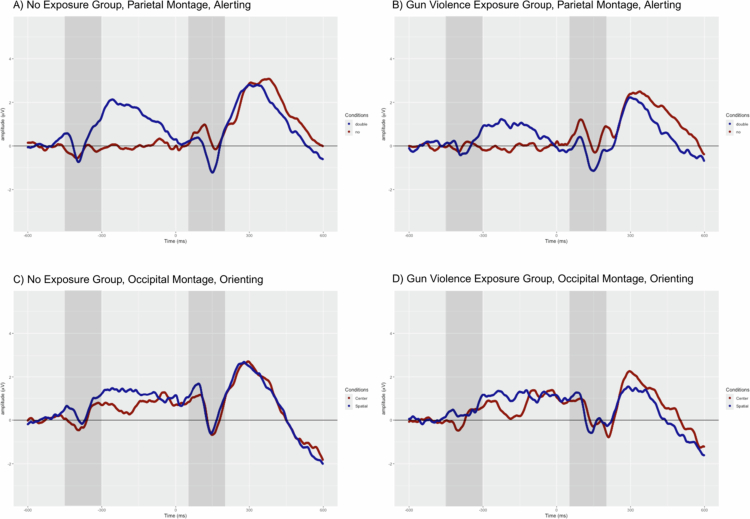
ERP waveforms for alerting and orienting conditions with cue-locked (−450 to −300 ms) and target-locked (50 to 200 ms) segments highlighted in gray. Responses averaged to generate alerting waveforms over the parietal montage with No Cue (red) and Double Cue (blue) trials for the **A.** NGVE group and **B**. GVE group. Responses averaged to generate orienting waveforms over the occipital montage with Center Cue (red) and Spatial Cue (blue) trials for the **C.** NGVE group and **D**. GVE group.

**Figure 4.**
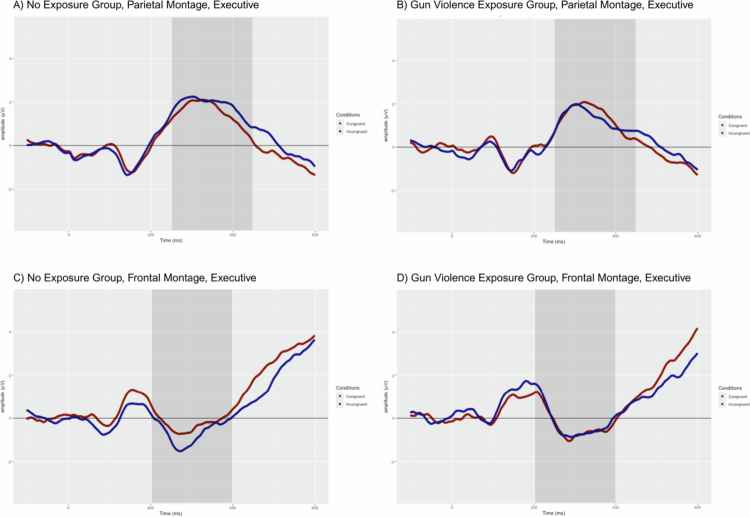
ERP waveforms for executive conditions responses from congruent (red) and incongruent (blue) targets. Target-locked segments of the P3 response (250 to 450 ms) from the parietal montage are shown for the **A.** NGVE group and **B**. GVE group. Target-locked segments of the N2 response (200 to 400 ms) from the parietal montage are shown for the **C.** NGVE group and **D.** GVE group.

Observing ERP responses after the presentation of the target stimulus allows for examination of the effects of cue type on the reaction to subsequent targets along with comparisons of congruent and incongruent target trials. Thus, we can assess differences within all three attentional systems: alerting (parietal N1), orienting (occipital N1), and executive (frontal N2 and parietal P3). Waveforms for the alerting and orienting conditions can be found in Figure 3; waveforms for the two executive control montages can be seen in Figure 4. Table 5 contains the mean peak amplitude for each of these components within each group.

**Table 5.** Target-locked peak amplitudes for cue conditions and target types.

		NGVE	GVE
Condition	Stimuli	*M (SD)*	*M (SD)*
Alerting N1**	No cue	−0.786 (.941)	−0.493 (.986)
	Double cue	−2.121 (.688)	−1.495 (.869)
Orienting N1	Center cue	−1.777 (.750)	−2.144 (1.042)
	Spatial cue	−1.985 (1.167)	−1.948 (1.149)
Executive control P3	Congruent target	2.518 (1.125)	2.207 (1.260)
	Incongruent target	2.788 (1.182)	2.056 (1.180)
Executive control N2	Congruent target	−.931 (1.529)	−1.185 (1.531)
	Incongruent target	−1.183 (1.830)	−1.824 (1.182)

Note: Mean and standard deviation reaction times are shown for the alerting, orienting, and executive control conditions for the NGVE and GVE groups. Times are shown in milliseconds.

^**^ Denotes significant interaction effect from LMM (*p* < .05).

Linear mixed models were computed to compare across groups and relevant trial types for each attentional system. Significant effects were only detected for the alerting N1 component. The LMM analysis revealed a significant interaction between Group and Condition (*t*(1152) = 1.974, *p* = .049). This interaction indicates that, although both groups showed differences between the trial types, the pattern was distinct for each group: the N1 difference was much larger for the NGVE group (No Cue: *M* = −0.786, *SD* = .941; Double Cue: *M* = −2.121, *SD* = .688) than for the GVE group (No Cue: *M* = −0.493, *SD* = .986; Double Cue: *M* = −1.495, *SD* = .869). The difference between the No and Double Cue trials for the GVE group was not significant (*t*(1152) = −2.096, *p* = .218); however, a significant difference between these two trial types was identified in the NGVE group (*t*(1152) = −5.220, *p* < .0001). In other words, the NGVE group demonstrated sharper differences between N1 amplitudes for the Double Cue versus No Cue trials as compared to the GVE group, which did not demonstrate significant differences between the trial types. A significant main effect of Condition was also found (*t*(1152) = 2.096, *p* = .036), indicating that, for all participants, irrespective of their GVE status, N1 amplitudes were larger in response to the Double Cue stimuli (*M* = −1.836; *SD* *=* .805) than to the No Cue stimuli (*M* = −.636; *SD* *=* .939).

Taken together, these findings suggest that differences in N1 amplitudes for No Cue and Double Cue conditions were identified across participants; however, these amplitude differences were largely driven by the NGVE group, particularly their outsized response to the Double Cue stimuli as compared to the No Cue stimuli. The GVE group largely demonstrated homogenous responses to these two types of stimuli. For the other attentional systems and components (orienting N1, executive N2, executive P3), no significant main effects or interactions were detected (all *p* > 0.05).

### Cue-locked analyses

For the cue-locked analyses, all time windows are relative to the onset of the cue stimulus instead of the targets. The components that follow these cues and precede the target help to index the direct effects of the cue type on specific attentional systems. We can therefore compare the immediate response to No Cue trials and Double Cue trials to assess the alerting system, and responses to Spatial and Central Cue trials to assess the orienting system. For each system, we examined two components: the cue-locked N1 (50−200 ms following the cue) and the cue-locked P3 (250−450 ms following the cue). As above, the alerting and orienting waveforms for each group are presented in Figure 3. Group average peak amplitudes are shown in Table 6.

**Table 6.** Cue-locked peak amplitudes for cue conditions.

Condition		NGVE	GVE
	Stimuli	*M (SD)*	*M (SD)*
Alerting N1	No cue	−.6816 (.4812)	−.4374 (.3451)
	Double cue	−.9969 (1.216)	−.9018 (1.002)
Orienting N1	Center cue	−.6974 (.8238)	−.6797 (.4859)
	Spatial cue	−.6707 (.8499)	−.1668 (.4879)
Alerting P3**	No cue	.5025 (.5900)	.3488 (.6275)
	Double cue	2.354 (.9565)	1.509 (1.118)
Orienting P3	Center cue	1.575 (.7238)	1.599 (.2961)
	Spatial cue	1.836 (.6772)	1.842 (.6104)

Note: Mean and standard deviation reaction times are shown for the alerting, orienting, and executive control conditions for the NGVE and GVE groups. Times are shown in milliseconds.

^**^ Denotes significant interaction effect from LMM (*p* < .05).

Significant effects in the LMMs were found only for the cue-locked P3 in the alerting conditions. There was a significant interaction between Group and Condition (*t*(1148) = −2.138, *p* = .033), indicating that, again, No Cue and Double Cue amplitudes differed in specific ways for the GVE and NGVE group. Though both groups showed a P3 effect, the difference in No Cue and Double Cue trials was much larger for the NGVE group (No Cue: *M* = .503, *SD* = .590; Double Cue: *M* = 2.354, *SD* = .957) than for the GVE group (No Cue: *M* = .349, *SD* = .628; Double Cue: *M* = 1.509, *SD* = 1.118). Pairwise comparisons confirmed that both differences between trial types were significant (GVE group: *t*(1148) = 3.107, *p* = .012; NGVE group: *t*(1148) = 6.367, *p* < .0001). Stated more simply, the NGVE group showed clearer differences between P3 amplitudes following the Double Cue versus No Cue trials as compared to the GVE group, which showed smaller differences between the trial types. A significant main effect of Condition was also found (*t*(1148) = −3.107, *p* = .002), which means that, across all participants, P3 amplitudes were significantly larger in response to Double Cue (*M* = 1.932; *SD* *=* 1.033) as opposed to the No Cue conditions (*M* = .426; *SD* *=* .607). However, no significant interactions or main effects were detected among any of the other LMMs for the cue-locked components (all *p* > 0.05).

## Discussion

In this pilot investigation, we applied neuroscientific methods to assess potential impacts of gun violence exposure on attentional processing using EEG and ERPs. Significant differences were found between the GVE and NGVE groups during the alerting condition of the ANT (*p* < .05). Alerting may be particularly impacted by traumatic exposures to gun violence due to its close links to hypervigilance, which can develop following the onset of trauma or in environments with a greater perception of threat (Smith & Patton, 2016; Smith et al., 2019). In addition, statistical analyses that consider individual-level differences, such as LMM, may be especially salient when studying gun violence, as the response to such a trauma is not homogenous. Empirically it can be difficult to make broad categorizations with a variable as sensitive and subjective as the processing of GVE.

Significant differences were not found in the orienting or executive control conditions, perhaps indicating that exposure to gun violence has specific effects on brain mechanisms associated with vigilance; however, larger sample sizes are needed to validate such interpretations. These results provide suggestive preliminary insights into how the alerting function may alter with exposure to gun violence. Considering the broader state of research on firearm-related harms, neuroscientific methods like these provide an opportunity to continue enhancing our understanding of the possible impacts of gun violence on the developing brain.

### The alerting response and hypervigilance

During the alerting conditions of the ANT, the Double Cue serves to ‘turn on’ the alerting system and prepare the participant to receive the next stimulus (Fan et al., 2002; Neuhaus et al., 2010). This preparation is indexed by the N1 component, and larger N1 amplitudes indicate increased processing of preparatory visual or other sensory information (Näätänen & Michie, 1979; Neuhaus et al., 2010). While the NGVE group showed significant differences in N1 amplitude between the No Cue and Double Cue conditions, GVE participants showed a more homogenous response to alerting-related stimuli, including smaller response differences between conditions with the cue-locked P3 (NGVE: *p* < .0001; GVE: *p* = .01) and no differences between conditions for the target-locked N1 (NGVE: *p* < .0001; GVE: *p* = .218). Theories of arousal and hypervigilance may provide one avenue for explaining these findings. Easterbrook's (1959) cue utilization hypothesis argues that physical arousal and attentional processing are inversely related: as arousal increases, the ability to concurrently process attentional cues in the environment decreases. Thus, in a state of high arousal, the attentional system is overtaxed, unable to take all environmental cues into account. Evidence has also shown that individuals with trauma-exposure and PTSD show heightened physiological arousal (e.g. Naegeli et al., 2018) while also being less likely to utilize environmental cues like spatial information (Barlow-Odgen et al., 2012; Russman Block et al., 2020). These effects on attentional resource allocation may stem from hypervigilance, which can enhance arousal via norepinephrinergic projections and bias the attentional system towards threat detection (Zawilinski, 2020). Taken together, these findings suggest that for individuals exposed to gun violence, the alerting system may be chronically engaged due to hypervigilance. Consequently, external cues that are not relevant to threat detection add little further activation (cf. Zawilinski, 2020), accounting for the observation of weaker target-locked N1 and cue-locked P3 effects.

### Methodological considerations

A central challenge in this study is the limited specificity with which participants’ histories of gun violence exposure can be characterized. Such exposure can vary along multiple dimensions, including frequency, recency, intensity, subjective impact, and results of physical or psychological harm. The relative weight of these experiences may differ substantially across individuals, and with a small sample it is difficult to adequately assess and categorize participants along such a breadth of factors. For this reason, we employed a simple binary classification of GVE, recognizing that this approach does not capture the nuance that larger samples may allow. To mitigate this limitation, we applied linear mixed modeling to increase the sensitivity of binary group comparisons by incorporating individual variability in ERP amplitude. Looking ahead, more detailed assessments of participants’ gun violence exposure histories, combined with statistical methods such as LMMs, Bayesian modeling, or regression approaches, will be important for parsing individual responses to these complex and subjective forms of trauma.

### Behavioral considerations

No significant group differences emerged for depression or emotional reappraisal, suggesting that the groups were homogeneous on these measures. However, the groups differed on emotional suppression, which reflects the habitual withholding of emotional expression (Gross & John, 2003), with the GVE group scoring significantly lower than the NGVE group. This finding runs counter to prior literature, which generally reports higher suppression scores with greater trauma exposure (Fitzgerald et al., 2017; Pugach & Wisco, 2023). One likely explanation for this is our small sample size, though it is also possible that emotional suppression may be more difficult to maintain for individuals with histories of gun violence exposure. Future research with larger samples should include a comprehensive assessment of mental health status to clarify these relationships.

### Limitations and future directions

This study has several limitations. First, its cross-sectional, non-randomized design reflects the practical and ethical impossibility of randomizing exposure to gun violence. Longitudinal work will be important to clarify whether exposure to gun violence produces sustained effects on neural processing of attention, similar to those observed following other kinds of trauma (see, e.g. Blair et al., 2015). Additionally, both gun violence exposure (Magee et al., 2022) and ERP amplitudes (see, e.g. Javanbakht et al., 2011) are associated with a range of mental health factors, underscoring the need for future work to comprehensively assess participants’ mental health histories alongside neural measures. Further, the small size and relatively homogenous racial composition (6 Asian; 6 White; 1 Hispanic or Latino; additional demographics provided in Table 1) of our sample was driven in part by challenges in recruiting participants willing to share their experiences with gun violence. Given that there are complex patterns of GVE differences across race (Semenza et al., 2025), income and urbanicity (Johnson et al., 2021), and gender (Kegler et al., 2022), a more heterogeneous sample would allow for an improved representation of the wide swath of experiences with firearm victimization. To more effectively recruit a diversity of participants, we recommend leveraging in-person recruitment approaches in partnership with community or advocacy groups, rather than the largely virtual outreach efforts that were employed for this exploratory study.

A significant barrier to this research was the absence of a standardized, quantitative tool for assessing gun violence exposure. The 14-item survey used here provided sufficient information for a binary categorization (no exposure/any lived exposure), but it did not capture important dimensions such as subjective impact, frequency, or recency of experiences. Future work should focus on developing more sensitive measures that more fully reflect the range of gun violence exposures in our communities. Objective indicators, such as the number of fatal homicides within a certain radius of participants’ homes (e.g. Gard et al., 2022), may provide valuable corroboration measures at the community level, and could be integrated with self-report questionnaires. Combining such measures with robust statistical methodologies would allow for more multidimensional categorization schemes, capable of isolating behavioral and neural effects linked to specific aspects of exposure. Similarly, modeling components of GVE as continuous rather than categorial variables may yield deeper insight into cumulative or long-term impacts. Finally, mixed methods designs incorporating qualitative approaches may more meaningfully capture the subjective impact of gun violence victimization. Whether applied through smaller approaches like coding free responses on validated surveys, or more in-depth methods like conducting semi-structured interviews (e.g. Ziminski et al., 2025), qualitative approaches may help researchers more holistically understand the impacts of gun violence on their participants.

This exploratory study represents the first empirical research (to our knowledge) that leverages EEG methods to elucidate the neurocognitive effects of violent firearm exposure. There exists a profound opportunity for interdisciplinary collaboration between neuroscientists and public health researchers to further define the neural sequelae of gun violence exposure, as well as any potentially adaptive responses to exposure to this form of adversity. Relatedly, future work should explore methods to connect cognitive evidence, such as the ANT, with day-to-day experiences for individuals with GVE. At present, it is inappropriate to extrapolate highly specified cognitive paradigms to daily life experiences; for instance, from this study, we can draw inferences about differences in the immediate processing of alerting-like stimuli, but it would be more speculative to extrapolate these findings into the participant’s daily experiences with attention. However, it is clear that downstream, changes to attentional mechanisms and aspects of hypervigilance can contribute to quality of daily life (Forbes et al., 2018). Interdisciplinary collaborations may therefore yield more inventive study designs which attempt to capture both neurocognitive effects and more tangible daily impacts on survivors of gun violence victimization.

## Conclusion

The present study used EEG and ERPs to examine the impacts of gun violence exposure on attentional mechanisms in late adolescents and young adults. Preliminary evidence suggests that exposure to gun violence may primarily affect the brain’s alerting system, as participants with such exposure showed greater similarity in neural responses to cue and target types associated with alerting. This pattern may reflect reduced differentiation in attentional responses, potentially linked to hypervigilance arising from gun violence exposure. The study was limited by a small sample size and by the use of brief measures of gun violence exposure and mental health. Future work should incorporate more comprehensive assessments of violence exposure, larger and more diverse samples, and longitudinal designs to better characterize these effects. Even with these limitations, the findings demonstrate that the ANT and its associated ERP components, especially those related to alerting, can be applied to study differences associated with gun violence exposure. Further, this exploratory study is one of very few probing the neural impacts of gun violence victimization, and the only one of which we are aware that used EEG and ERPs to investigate the phenomenon. Neuroscientific approaches offer a valuable perspective on how such exposure may shape brain and behavioral functioning, reinforcing the urgency of addressing gun violence as a threat to adolescent development.

## Data Availability

The data that support the findings of this study are available from the corresponding author, EFE, upon reasonable request.

## Impact statement

This empirical study piloted the application of a neuroimaging approach, event-related potentials (ERPs) derived from electroencephalography (EEG), to investigate the impact of gun violence exposure on the neural mechanisms of attention. Individuals with greater exposure demonstrated evidence of reduced efficiency in the alerting component of attention, which may reflect hypervigilant or overtaxed attentional systems resulting from traumatic experiences. Because neuroscientific approaches have rarely been used to examine the causes and consequences of violent firearm exposure, this study provides preliminary evidence for the feasibility of using EEG to investigate these processes. These findings may also facilitate future collaborations between neuroscientists and public health specialists to better understand the neurophysiological effects of gun violence exposure.

## Abbreviations

GVE: Gun Violence Exposure
NGVE: No Gun Violence Exposure
ANT: Attention Network Test
EEG: Electroencephalography
ERP: Event Related Potential
PTSD: Post-Traumatic Stress Disorder
fMRI: Functional Magnetic Resonance Imaging
BDI-I: Beck Depression Inventory-I
ERQ: Emotion Regulation Questionnaire
SECV: Survey of Exposure to Community Violence
LMM: Linear Mixed Model

## Appendix 1

**Table A1.** Community experiences.

	All		NGVE		GVE	
Experience	*n*	%	*n*	%	*n*	%
Gun Violence Exposure						
Heard gunshots in neighborhood	6	46	0	0	6	100
Carried firearm due to feeling unsafe	0	0	0	0	0	0
Close friend shot with firearm	0	0	0	0	0	0
Family member shot with firearm	1	8	0	0	1	17
Witnessed someone get shot with firearm	2	15	0	0	2	33
Experienced school lockdown due to threats	3	23	0	0	3	50
No gun violence experience	7	54	7	100	0	0
Community Violence Experiences						
Witnessed someone else getting beat up	4	31	3	43	1	17
Witnessed someone else get stabbed	0	0	0	0	0	0
Heard people talking about having weapons	9	69	3	43	6	100
Seen other people carrying weapons	2	15	0	0	2	33
Seen other people threatening to hurt others	8	23	4	57	4	67
Seen other people get hit or pushed	7	54	4	57	3	50
Been hit or pushed	3	23	1	14	2	33
Received threats of harm	5	38	1	14	4	67
Seen someone break property on purpose	0	0	0	0	0	0
Positive Neighborhood Events						
Seen people helping each other with house or yard work	12	85	6	86	6	100
Been helped by someone	13	100	7	100	6	100
Recency of (all) community events						
Within the last year	6	46	4	47	2	33
1−5 years ago	3	23	0	0	3	50
5−10 years ago	3	23	2	28	1	17
10−15 years ago	1	8	1	14	0	0

Note: Participants’ community experiences (number and percentage), including gun violence exposure, community violence exposure, positive community experiences, and recency of experiences, are shown for all participants, the no gun violence exposure (NGVE) group, and the gun violence exposure (GVE) group.
